# Co‐Designing a Social Media and Anxiety Survey: Reflections on the Importance of Centring Mental Health Lived Experience Expertise

**DOI:** 10.1111/hex.70677

**Published:** 2026-04-24

**Authors:** Sharon Lawn, Kerri M. Gillespie, Aakanksha Sahu, Stephanie J. Tobin, Vasundhara Shukla, Rachael Burns, Joanne Cockle, Amrita Dasvarma, Aislin Gleeson, John Milham, Robyn Priest, Puneet Sansanwal, Ashton Spradbury, Anna Zhang, Christine Kaine, Selena E. Bartlett

**Affiliations:** ^1^ Lived Experience Australia Adelaide South Australia Australia; ^2^ Flinders University Adelaide South Australia Australia; ^3^ School of Clinical Sciences, Faculty of Health Queensland University of Technology Brisbane Queensland Australia; ^4^ Translational Research Institute, Faculty of Health Queensland University of Technology Brisbane Queensland Australia; ^5^ Mater Research Institute The University of Queensland Brisbane Queensland Australia; ^6^ School of Psychology and Counselling, Faculty of Health Queensland University of Technology Brisbane Queensland Australia; ^7^ All India Institute of Medical Sciences Ansari Nagar New Delhi India; ^8^ SAGE‐DM Lived Experience Advisory Panel

**Keywords:** anxiety, co‐design, lived experience, mental health, social media

## Abstract

**Introduction:**

This study explores a collaborative co‐design process undertaken with people with lived experience expertise (PLEE), to develop a survey investigating experiences of social media and anxiety. The research is the first step in a larger project across five countries (Australia, the United Kingdom, India, Canada and Singapore) that will seek to validate whether passive smartphone analytics and codesigned ethical protocols can underpin a scalable culturally inclusive AI chatbot that detects and mitigates anxiety based on smartphone use.

**Methods:**

Through three iterative co‐design workshops, conducted in Australia, facilitated by and involving people with lived experience of mental health conditions, insights were gathered on psychological, social, and structural mechanisms by which social‐media use influences anxiety.

**Results:**

Co‐design workshop members strongly challenged the research team within five important themes: (1) reframing risk and safety that involved ‘calling out’ disempowering and discriminatory language inherent in survey processes and existing validated measures; (2) social media as both harm and Haven that emphasised social media as both a source of anxiety and a lifeline for connection for this population; (3) designing for inclusion, accessibility, and safety to ensure survey usability and psychological safety for future participants; (4) transparency, power, and representation to ensure lived experience involvement meant shared ownership, avoided tokenism, included First Nations leadership; and (5) broadening the lens – cultural, physical, and socio‐economic factors involved urging a holistic view of the person and a systems view of anxiety and technology.

**Conclusion:**

By involving people with mental health lived experience expertise in the design process, this study was able to co‐create recommendations to strengthen the project's survey design, ethical framework, and implementation plan. The co‐design approach ensured the social media and anxiety survey met the specific needs of the target group and was trauma‐informed, promoting trust, engagement and feasibility. Future research will aim to focus on gathering insights from similar lived experience co‐design workshops in the United Kingdom, India, Canada and Singapore to refine the AI Chatbot prototype and evaluating its effectiveness in a broader study. This study underscores the crucial role of mental health lived experience expertise in research that seeks to test digital solutions for people who experience anxiety exacerbated by social media use.

**Patient and Public Contribution:**

People with lived experience of a mental health condition contributed throughout the design, analysis and write‐up of this work as members of a Lived Experience Advisory Panel (LEAP) which met over a series of co‐design sessions. The co‐design was led by a mental health lived experience researcher who was also a key member of the research team for the larger project. They led the reflexive thematic analysis, and writing and reviewing of the manuscript, in partnership with the co‐design group members and the wider research team. Whilst some members of the academic research team identified as having mental health lived experience, they did not undertake their research roles from this perspective.

## 1 Introduction

Anxiety is common across multiple mental health conditions, co‐occurring with conditions such as depression and schizophrenia. It is a leading mental health concern among children, young people, and adults, and evidence increasingly identifies social media as one main driver for its development and maintenance [1]. Fear of judgement, compulsive scrolling, and distress after online interactions [2], plus repetitive social media use to help manage loneliness, social isolation, and low self‐worth [3, 4, 5] are known contributors to anxiety. However, social media use can also reduce anxiety [6], and there appear to be differing impacts on anxiety between passive and active use, as well as positive and negative experiences of social media, that require further research [5, 7].

Most research on the impacts of social media relies on self‐reported screen time or pathological use, which may miss the real‐time context in which emotional nuances can be expressed at the time the person is using social media. Much of the existing research also focuses on internet addiction or gaming addiction [8]. Also, the potential intersecting relationships between screen duration, type of social media content, purpose for using social media, and emotional context are under‐researched. Current digital interventions may therefore fail to detect distress in time [5, 9]. Consequently, interventions that may be developed to offer information, self‐agency and support in real time, as the person is using social media, are lacking. Co‐design with PLEE is well‐placed to explore and address these research gaps because they undertake and experience, firsthand, the daily processes involved in using social media, and are the most directly impacted by using social media. Through their positioning, they possess a type of lived experience knowledge and expertise that can only be seen, felt, heard and understood from direct lived experience.

Governments are increasingly challenged by the communities they serve to deliver effective policies and funding across multiple competing priorities such as health, defence, education, welfare and climate change. Likewise, communities have become increasingly engaged with questions about what constitutes expert knowledge and evidence, important for understanding and addressing society's complex problems. One consequence of these shifts is increasing pressure to conduct research that is fiscally responsible, equitable and accountable, and that has genuine translational value and impact for communities as well as return on investment for governments. This has contributed to the elevation of participatory research methods such as co‐design with communities. Demonstrating the active involvement of people with lived experience expertise (PLEE) beyond their role as ‘participant’ is now a formal requirement for many research grant schemes internationally [10, 11, 12, 13].

Evidence for the value of involving PLEE in improving research quality, recruitment, rigour, outcomes and dissemination is well‐established [14, 15, 16]. It also leads to greater feelings of empowerment among communities being researched, particularly more marginalised, disadvantaged and stigmatised groups, as well as greater researcher insight into and rapport with those communities [15, 17]. Co‐design has become the basis of doing research ‘with’ rather than ‘on’ individuals and communities. Based on Arnstein's ladder of participation [18] involvement of PLEE is now considered a core criterion for doing effective research, noting that it sits along a continuum (from consultation, involvement, co‐design participation, to coproduction) that reflects increasing levels of partnership and power sharing [19]. PLEE and their community organisations have demonstrated that they possess unique expertise in transforming social and health services to improve social, economic and health outcomes for affected communities [16]. Co‐producing research, which includes people at the heart of the research interest, has distinct advantages in creating more appropriate recruitment strategies, embracing compassion for the participants by acknowledging their experience, ensuring the process is supportive, the content meaningful, and also contributes to study success [15, 20]. The resulting benefits of the sharing of power and knowledge have been suggested as improving health services and outcomes through increased attunement and suitability of programmes to cultural and logistical contexts, empowering communities, and the facilitation of knowledge translation into practice [21, 22]. However, authentic involvement of PLEE in research continues to be challenging to implement due to a range of factors such as researcher knowledge, skills, confidence, and attitudes. This includes a lack of recognition that PLEE have valid forms of knowledge to bring to the research process; that they have expertise that is inherent in their experience [23, 24].

Whilst lived experience perspectives and leadership in research exist, study numbers remain low, with few reflecting on or espousing best practice, and noting difficulties identifying the epistemological impact on the research process and outcomes [25, 26, 27]. In an Australian study [26], p.6), members of the project stakeholder group and the academic researchers “found the coproduction process to be transformational with learnings on how the sharing of power and governance through the research relationship enabled a sensitivity and responsiveness in approaches to knowledge creation. The process was at times referred to as therapeutic, highlighting the presence and knowledge of people with similar experiences being able to hold spaces safely [to allow vulnerability and safe sharing] and recognising the need for appropriate use of language in drafting research questions” (see also [28]). An Australian study with mental health family carers by Walters et al. [26] found the principles that promoted partnership were: lived experience leadership and inclusion from the beginning, power sharing within the research partnerships, researching with people and not on or about them, valuing the process of coproduction in research through time and resource allocation, and recognising and valuing lived‐expertise and coresearchers within research processes.

Co‐production is particularly important and useful in the field of mental health research, where social justice concerns can be significant, and where the intersection of multiple complex issues can be common. The involvement of PLEE is crucial to more fully understand potential causes and solutions [29]. This is because PLEE must navigate the contradictions, complexities, and intersections (such as biopsychosocial influences and impacts, and complex systems and relationships) that can make living with mental health challenging, and for which more holistic approaches that address many intersecting factors are needed.

One area of growing concern within the mental health field is the impact of social media on anxiety. Anxiety can be understood as a person's ‘normal’ response to the stress or worry of everyday life; how we naturally react when stressed and is characterised as being temporary and not harmful in the long term. “It warns us of possible dangers ahead. We feel nervous or worried for a bit, especially when facing certain situations or events. It's crucial for getting us ready for challenges” [30]. Conversely, if the anxiety does not go away, this can make it hard for people to function, feel much heavier, and make daily life hard. It can develop into a mental health condition that may persist and not resolve without provision of other support and/or treatment [30].

This study therefore involved workshops with PLEE of mental health conditions to co‐design a survey to determine which social media data features are most relevant and under what contextual conditions these features become meaningful for identifying anxiety. This co‐design phase was undertaken to foreground a larger study of social media and anxiety (SAGE‐DM) as part of a Wellcome Trust Accelerator Program, with the eventual goal to co‐design and validate a culturally inclusive AI Chatbot to detect and mitigate anxiety based on social media exposure.

## 2 Materials and Methods

### 2.1 Design

This project used participatory methods that reflect Arnstein's [18] “Degrees of citizen power” to promote collaborative governance between PLEE and academic researchers [21]. Reflections on the project's process of co‐design utilised a narrative approach to illustrate the process for knowledge generation [31].

### 2.2 Lived Experience Co‐Design Participants

The SAGE‐DM Lived Experience Advisory Panel (LEAP) included people with lived experience of anxiety and social‐media use challenges. The SAGE‐DM (Social media, Anxiety, Guidance, and Ethics using Data‐Driven Mechanisms) project aims to identify and reduce the behavioural and emotional mechanisms linking social media engagement with anxiety. The intersectionality of their lived experience included identifying as mental health service users, family and carers, systems advocates, and lived experience researchers. Diversity of age, gender, and cultural identity was emphasised. Ten LEAP members were recruited. Of these, seven were recruited through Lived Experience Australia (LEA), a national lived experience systemic advocacy and research organisation with a ‘friends’ network of more than 12,000. Within this large network sits a dedicated group of over 120 lived experience representatives with diversity across mental health conditions and experiences, geographies, and identities who are engaged iteratively as research advisors, focus group participants, and co‐creators across LEA's systemic advocacy and projects. Expressions of interest, selection and matching of lived experience representatives occurs via LEA's Customer Relations Management (CRM). For this project's selection criteria, representative panel members were asked to briefly describe their motivations for seeking involvement in the project, their lived experience of social media use and anxiety, and their availability to participate in the project. The project's lived experience lead reviewed the expressions of interest, seven (from 10 who applied) which met all three criteria. Three further LEAP members, who also met the three selection criteria, were approached directly by the project's lived experience lead because of their known lived experience research knowledge, and their linkages with culturally diverse mental health lived experience communities in Australia, and understanding of social media/technology usage in India, Singapore, the UK and Canada. This diversity was important, given the larger project plan to connect with lived experience networks and establish further LEAPs in Canada, India, Singapore, and the UK.

### 2.3 Co‐Design Process and Facilitation

The LEAP was convened in September 2025 to co‐design and review the ‘Social Media and Anxiety’ survey instrument, forming the foundation of the SAGE‐DM project. Their involvement guided the development of survey research questions, data interpretation, ethical frameworks, and dissemination strategies. Three 2‐hour sessions, approximately 1 week apart, engaged the 10 PLEE members. Sessions were online for convenience, given LEAP members were located across Australia. The LEAP was facilitated by the project's lived experience research lead (SL). Due to their dual role as an academic researcher on the larger SAGE‐DM project and also as a lived experience researcher, the LEAP facilitator took steps before, during and in‐between each LEAP meeting to reflect on their positioning as a figure with greater potential power than other members of the LEAP. These steps included striving for transparency with LEAP members in all documentation brought to and arising from the group discussions, having an open agenda contributed to and then driven by the LEAP discussions (not the academic team), debriefing with LEA lived experience peers, and connecting with LEAP members individually outside of the sessions to seek feedback on their participation experience. They also shared and modelled the ethos of the LEA ‘Shared Advocacy Spaces’ commitment statement throughout the project (https://www.livedexperienceaustralia.com.au/safe-research-partnerships), and this also set the tone for equity and inclusivity of contributions by LEAP members during the sessions.

The overall SAGE‐DM project academic lead (SB) and senior research officer (KG) attended the first session to briefly introduce the project, introduce themselves to LEAP members, welcome lived experience members and thank them, then left the meeting so that LEAP members could speak freely. This was intentional, to foster safety in the discussions and ensure any perceived or actual undue influence, dominance, or bias from the research team was minimised [32]. The LEAP discussions aimed to:

- Ensure that lived experience voices guide ethical, inclusive, and culturally sensitive research design.
- Identify psychological, social, and structural mechanisms by which social‐media use influences anxiety.
- Co‐create recommendations to strengthen the project's survey design, ethical framework, and implementation plan.

LEAP sessions were iterative, each building on the previous session, with an open agenda for the main section (the discussion to determine and refine the survey), determined by the LEAP. This ensured focused discussions [33], pace and priorities were set by the LEAP, and that each next session picked up from where the group had reached in the previous session and reflected the time they needed, without pressure. LEAP sessions were audio‐recorded and transcribed, with permission, to ensure accurate capture of the discussions. Group interactions were guided by a broad set of open‐ended and closed questions (Supporting Information S1: File 1). The session outlines were as follows:

- **Session 1:** Orientation to SAGE‐DM, open reflection on social media and anxiety, and preliminary discussion of survey scope.
- **Session 2:** Detailed review of the draft survey; language, order, tone, and accessibility.
- **Session 3:** Reflection on process, survey usability, and ethical principles for co‐design.

### 2.4 Data Analysis

Each session was transcribed verbatim, de‐identified by the LEAP facilitator prior to sharing with research team members and then analysed thematically using reflexive thematic analysis [34]. This approach emphasises the researcher's self‐awareness and critical reflection throughout the analysis process, paying particular attention to power dynamics, and maintaining a transparent audit trail of decisions made during the research process. Its intention is to explicitly account for the researcher's influence, perspectives, and assumptions on interpretation of the data, in order to enhance depth and credibility of the analysis and improve overall rigour and transparency [34].

The LEAP facilitator and two academic members of the research team (each with family or personal lived experience of mental health challenges but not working in dedicated lived experience research roles or bringing a lived experience‐informed perspective to the analysis) undertook the reflexive thematic analysis. This involved independent coding and note‐taking, then robust group discussion. They then presented their critical reflections and draft summary of ideas to the LEAP members for further discussion, reflection, and finalisation. There was strong alignment with the tentative themes with few changes suggested by the LEAP ‐ theme 4 arose from the LEAP discussion and likely signified the equity and safety that LEAP member felt in expressing their perspectives.

The analysis involved the following steps:

- Familiarisation: Reading and re‐reading the de‐identified transcripts from the three LEAP sessions, to become immersed and intimately familiar with their content.
- Initial reflection: Noting initial observations and insights from specific segments of the co‐design group discussions that stood out (e.g. because they were perceived as unexpected, challenged existing understandings), in relation to the entire dataset, and meeting to discuss these insights.
- Coding and generating initial themes: Generating succinct labels to capture and evoke important features from the LEAP discussions that were identified from the initial reflections process, to begin to develop significant, broader patterns of meaning (potential themes).

- Refining, defining and naming themes: Research group discussion to develop each theme, its scope and focus, determining the ‘story’ of each and how it sat within the overall narrative.
- Write up, PLEE discussion, finalisation: Weaving together the draft analytic narrative and data extracts from transcripts, then presenting these to the PLEE for discussion and revision, as needed, before finalising the analysis.

## 3 Results

Five themes are presented here, reflecting key insights from the analysis of LEAP discussions. For each theme, a brief description is provided, with de‐identified exemplary quotes from LEAP participants. This is followed by brief practical implications arising from the theme insights, for the planned survey and further study steps. All voices from the LEAP members were represented equally in the results; no single person's opinion was privileged. Quote‐related demographic information was not provided in order to preserve privacy and anonymity of comments.

### 3.1 Theme 1: Reframing Clinical Diagnosis, Risk and Safety

LEAP participants strongly challenged the research team to think beyond clinical pathology and diagnostic labels, to more holistic understandings of mental health challenges. This was particularly noted during the discussion of the draft survey's demographic questions, which initially listed several diagnoses to select from. LEAP participants emphasised that this reflected a traditional researcher‐driven approach that positions and labels people as objects of research. They stressed the importance of a person‐first approach that maximised the person's empowerment and agency.

I don't identify with any of the diagnoses that I was treated for….I just wanted to query a bit more what the purpose is of people saying what diagnosis they've got…maybe it's more about asking if people identify as having a mental health issue or have interacted with the mental health system. And then you might get people who say, no, I've never had anything to do with mental health. That's not something I identify with at all…we're never going to have a comprehensive list. And also, then it just places a bit more, I suppose using that word agency for people to engage with the survey in the way that they feel comfortable to do so and disclose the information…having a list format can be quite limiting. And then it also stops people providing information that also might be quite relevant that we haven't thought of.

Another specific example was the LEAP participants' discussion of the inclusion of standardised body image questionnaires in the draft survey. They voiced strong opposition to the narrow clinical focus on body image concerns, stressing that they were too closely related to eating disorder concerns.

This looks very similar to an eating disorder assessment. I personally would just say that I'm not a fan of the real heavy focus on the link between like body image concerns and social media… there's so much representation in that already. And my fear is that in a world in which we need more research in eating disorders and the relationship between eating disorders and other causal factors, I feel that this would then be used in a way that reinforces the idea that eating disorders are purely body image concerns.

Participants also strongly challenged the research team to rethink the framing of *“risk.”* They viewed risk as contextual and relational, not simply as exposure to harm.

I'm very passionate talking to in that we need to allow people to take these risks to learn…a lot of the time if it's someone that doesn't have a history of mental health challenges, we'd be so OK having an amount of risk and it feels like there's often kind of like a better word, kind of like cotton wool, like wrapping in cotton wool around people, which is a form of discrimination.

The concept of “dignity of risk” was proposed – that people with lived experience should have equal power in deciding what constitutes safe or unsafe engagement.

If people are not in the room and risk is being spoken about them, that's not collaboration. When the person is in the room with equal power, that's when it's ethical.

Several contributors noted that over‐protective or paternalistic approaches can be disempowering and discriminatory. At the same time, participants emphasised the *structural risk* of profit‐driven algorithms and manipulative platform design.

I think that we have to understand that the providers of social media are not our friends. They're bad actors. They have an agenda that is not designed to support. And I think when you factor risk in, dignity of risk is wonderful, right? But we're talking about us versus the algorithm…We're up against the algorithm, not each other… their agenda isn't our wellbeing.

#### 3.1.1 Practical Implications

- Switch demographic questions about diagnoses from being a pre‐determined list to asking the person to self‐identify (person‐first, empowerment, agency).
- The survey and subsequent chatbot design must *balance protection with autonomy*.
- Include explicit recognition of user agency and the right to informed digital participation.
- Avoid language that positions users as passive, ‘at‐risk’ or fragile.

### 3.2 Theme 2: Social Media as Both Harm and Haven

Across all three panels, LEAP participants articulated a *dual experience* of social media — as both a source of anxiety and a lifeline for connection. They described doom‐scrolling, cyber‐bullying, and body‐image pressures, yet also highlighted online communities that provided belonging and advocacy opportunities.

It's the first time I've ever felt in community and confident to advocate. That's life‐changing.

I rely on social media to stay connected with family overseas, but it can also raise my anxiety when I read distressing news.

This ambivalence prompted a recommendation that the survey explicitly allow *simultaneous positive and negative responses*, reflecting the duality that can be present in their lived experience, rather than forcing binary choices. Participants also noted how experiences varied depending on mental‐health state:

If I'm running well, social media helps. If I'm not, it makes everything worse.

#### 3.2.1 Practical Implications

- Reword survey items to capture *ambivalent experiences* (e.g., “both helpful and stressful”).
- Include open‐ended text boxes to allow personal nuance.
- Ensure final chatbot design acknowledges this emotional complexity.

### 3.3 Theme 3: Designing for Inclusion, Accessibility and Safety

A recurring thread concerned survey usability and psychological safety. LEAP participants stressed that completing an anxiety‐related survey about social media could itself be *anxiety‐provoking*. They advised including a compassionate preamble outlining what to expect, normalising emotional responses, being sensitive to the use of language within the survey, and providing opt‐out options.

You're asking someone with social‐media anxiety to do a social‐media survey — that's anxiety‐raising in itself. There needs to be a strong preamble and focus on safety.

Whilst the researchers' everyday use of the word ‘tracked’ to describe features for monitoring social media usage, the LEAP stressed that this word was triggering and could increase anxiety in the populations being researched because it had connotations for their lived experiences of coercive systems, control issues, and breaches of social justice and human rights.

Look, since COVID, nothing that has the word tracked or observing can fly well, just because, yeah, we had, especially Victorians…that inhumane 650 days of lockdown and being observed, being tracked, this and that. Yeah, it's just any sort of authoritarian involvement with human beings is, yeah, won't fly well.

Other design feedback:

- Use plain, conversational language: replace “mood” with “changes you notice”.
- Make questions optional and allow return to incomplete surveys.
- Avoid a clinical tone that might evoke diagnostic testing.
- Add First Nations identity as a separate demographic question, not merged into “other communities”.
- Consider neurodiversity and accessibility, e.g., clarifying ambiguous time frames (“more than half the time”) and reducing survey length.

#### 3.3.1 Practical Implications

These recommendations significantly informed revisions to the pilot survey and ethics documentation.

### 3.4 Theme 4: Transparency, Power and Representation

LEAP participants highlighted that true co‐design requires shared ownership of interpretation. They proposed that the *demographics of the research team* be transparently reported – for instance, including the age range or lived‐experience background of analysts.

If you're asking participants their age, show them the ages of the people analysing the data. It builds trust.

They also called for:

- First Nations leadership on the project from inception, not post‐hoc inclusion.
- Recognition of carers' perspectives as *distinct lived experience*, not secondary data sources.
- Equal respect for emotional and intellectual expertise: lived experience was framed as *professional knowledge*.

I'm not here to be triggered – I'm here as a professional expert through experience.

#### 3.4.1 Practical Implications

- Include lived‐experience co‐authors and analysts on all outputs.
- Embed transparent authorship and data‐interpretation statements in reports.
- Continue co‐design into the AI and chatbot development phase, not just the survey stage.

### 3.5 Theme 5: Broadening the Lens – Cultural, Physical, and Socio‐Economic Factors

Participants consistently urged a *systems view* of anxiety and technology. They linked algorithmic design, profit motives, and socio‐economic pressures:

Social media, AI, internet, more than it's a resource, it is a profit‐making, money‐making, revenue generating, system machinery – advocacy here faces an extra hurdle.

They also drew attention to *physical* and *embodied* impacts of social media: sleep disturbance; eye strain; posture; nutrition; and lack of exercise. Culturally, they recommended including non‐Western and non‐mainstream platforms (WeChat, Telegram, BlueSky, Reddit and Discord) to ensure global relevance.

LEAP participants also highlighted the importance of broadening the focus on impacts of social media use and anxiety beyond ‘while you are using social media’. They emphasised that anxiety from social media use, particularly use for people with mental health challenges, can have residual impacts (such as feelings of guilt, shame, worry, low self‐worth) that extend well beyond and after the actual social media use; that these are distinct processes and experiences that should be measured separately.

Another example was the draft survey's focus on social media use and anxiety only within the personal world of the person, arguably reflecting a siloed focus on individual pathology and assumptions about the populations to be researched. LEAP participants reminded the researchers that social media anxiety could also be related to social media use at work.

I have more anxiety about my work social media because of the expectation that I will do it more often. Whereas personal, I'm like, yeah, I'll just throw something on here and I have no anxiety about that at all…There's a bit of FOMO on that too…I should because others are doing it. So, I'm now afraid that I'm missing out.

#### 3.5.1 Practical Implications

- Ensure culturally diverse platform examples across survey versions.
- Amend surveys based on LEAP feedback in the specific country and country‐based contextual factors.
- Integrated new sub‐scale on sleep, posture, and physical well‐being into the survey.
- Integrate somatic and lifestyle indicators in mechanistic model design.
- Consider equity issues in future recruitment, ensuring access for lower‐income or digitally excluded populations.
- Consider the life‐course of social media use and anxiety, not only measurement of the momentary use of social media.
- Added item distinguishing anxiety related to personal versus professional use of social media.
- Recognise other uses of social media, for example, as a tool for finding information on how to do things (e.g., wedding preparation, house hunting, etc.).

## 4 Discussion

The themes derived from the co‐design discussions reported in this study provide a powerful example that demonstrates the value of lived experience collaboration in research. Many of the issues raised by the LEAP were ‘blind spots’ that the researchers may otherwise have not realised without the involvement of lived experience expertise. In the current project, PLEE were invited to participate in discussions alongside academic researchers to reflect upon the project's co‐design process. The LEAP findings directly informed revisions to the Phase 1 proposal and pilot survey that will foreground the broader SAGE‐DM project. These modifications position the project as a leading model of ethical, participatory AI design in mental‐health research. While the broad position and focus of the current study are most appropriate for use in Australia, findings such as those noted in themes 3 and 4 about first people/nations leadership exemplify areas within the framework that can be applied for exploration in other countries.

Mental health experiences and help‐seeking behaviours are shaped by cultural and social context. Research involving people with lived experience across diverse settings shows that factors such as stigma, family expectations, and trust in health systems can influence how individuals engage with support and with research participation [35, 36]. These considerations highlight the importance of culturally responsive approaches when developing digital mental health tools. Exploring these issues more deeply across culturally diverse populations will be an important focus of future work.

In their systematic review of published frameworks supporting patient and public involvement in health‐related research [37], identified 65 frameworks. Thirty of the frameworks (13 power‐focused (changing or disrupting traditional power relationships) and 17 partnership‐focused) were reviewed, to identify underlying principles that would support partnership in research. Principles that promoted partnership included public‐led governance, transparent processes, clear communication, accountability, discussion about roles, reimbursement (fairness of opportunity), respect, responsiveness, support, training, and capacity building [37]. Extending on these ideas, several lessons were learned from the current study:

1. Lived experience must shape methodology, not only content.
  – The LEAP influenced language, structure, and ethical framing, not just question items.
2. Safety emerges from collaboration, not control.
  – Co‐designing with lived experience experts creates a more genuine safeguard than imposing pre‐defined risk frameworks.
3. Ambivalence is data.
  – Both positive and negative experiences hold equal scientific value and must be captured in analysis.
4. Representation matters.
  – Visible diversity in research teams builds trust and cultural validity.
5. Process is pedagogy.
  – The act of co‐production itself educates researchers, transforms relationships, and models the empathy central to digital‐mental‐health innovation.

Findings showed that the LEAP influenced conceptual and methodological decisions, rather than simply changing or refining questionnaire items. Where amendments occurred, the rationale frequently cited LEAP feedback on accessibility, autonomy, and safety, indicating that lived experience input had altered the study's structure and participant‐facing processes. Analysis of the LEAP meeting notes demonstrated changes to language: emphasising accessible and empowering language and challenging our understanding of accepted terminologies. Participants also highlighted issues relating to questions that required black‐and‐white answers, when the question was open to ambivalent and multifaceted responses that could provide greater context and meaning. LEAP contributors suggested safety practices perceived as more acceptable and practicable than the pre‐specified risk frameworks imposed by entrenched scientific methodologies. Findings suggest a potential for greater adherence to safety protocols that are developed collaboratively with lived experience expertise.

Ambivalence was evident in the LEAP discussions, with participants expressing both interest in tools that could support their well‐being and caution about how such technologies might be used or interpreted. This pattern is consistent with research involving people with lived experience of mental health challenges, which shows that ambivalence is a common and understandable part of help‐seeking and recovery. Individuals often simultaneously hold a desire for change while also experiencing uncertainty related to stigma, privacy, loss of control, or previous negative experiences with services. Behavioural change frameworks such as motivational interviewing emphasise that ambivalence should not be viewed as resistance, but rather as a normal part of the change process that can be explored through respectful and collaborative engagement [38]. Involving people with lived experience in the design of digital mental health tools may therefore help address these concerns by increasing transparency, trust, and user control [39, 40].

Transparency was considered a crucial aspect of research, which is often lacking, but essential to build the trust and rapport necessary to ensure study adherence and honesty in reporting. Reflexivity in research (the process of critically examining and acknowledging one's own biases and influences) has been embraced in qualitative research but is rarely incorporated or reported in quantitative research. Even in qualitative studies, reflexivity is often poorly understood and superficially discussed [41]. LEAP participants took it a step further, requesting additional demographic information about the researchers to gain a better understanding of the research team. LEAP members also emphasised the importance of avoiding a narrow research scope, which can unnecessarily limit findings and impair the successful interpretation and generalisability of research results. Factors investigated in a research project may impact many aspects of a person's life and may, in turn, be impacted by numerous other personal and lifestyle factors.

Framing research with mental health populations through an empowerment and human‐rights lens challenges the longstanding deficits‐focused paradigm that risks othering participants and reducing lived experiences to symptoms or dysfunction. The approach suggested by LEAP members centres on agency, dignity, safety, and acknowledgement of the expertise of individuals with lived experience, shifting the focus of research from observing ‘subject’ to collaborating with partners. These lived experience partners can reveal strengths, mechanisms, and contexts often invisible to purely scientific or clinical frameworks. By embedding empowerment into study design, recruitment, consent, data collection, analysis, and dissemination, research becomes not only more ethically defensible but also better equipped to produce meaningful and effective outcomes.

Promoting a more holistic research agenda counters the narrow foci that may perpetuate bias and limit our understanding of solutions to complex social issues, such as social‐media‐related anxiety. Because it is difficult to isolate the effects of social media from the many interlaced social, developmental, psychiatric, and environmental factors that confound all human experiences, our initial work deliberately tests correlations and feasibility within a constrained scope. This narrow focus is a pragmatic first step to establish a defensible relationship that we can later expand upon. As one participant observed, capturing every relevant variable is impossible, so rather than attempting an unattainable model, we foreground a consumer‐driven, nuanced approach that identifies the lived experiences of social‐media‐related anxiety with increased ecological validity. LEAP‐informed research, by prioritising context, participant voices, and lived experience insights, moves beyond the narrowly focused, methodical approaches of clinical models, yielding findings with greater real‐world relevance and applicability.

### 4.1 Reflections on Process

Across all sessions, participants praised the *quality of facilitation* and sense of psychological safety.

I've loved the process – it's broadened my horizons and improved my confidence.

There was gold in the unscripted conversations.

The meetings fostered mutual learning and challenged hierarchical assumptions between researchers and lived‐experience experts. Participants expressed that the process itself modelled *the change they want to see in research*: relational, transparent, and co‐creative. However, logistical challenges included time limitations, online fatigue, and the emotional labour of sustained engagement. Future sessions will benefit from shorter, more frequent interactions and compensation models recognising professional expertise.

### 4.2 Strengths and Limitations

The inclusion of PLEE into the conceptualisation, design, and implementation of research has been conducted to improve the safety, acceptability, and success of research. However, opinions are likely to be diverse, particularly across such a wide range of individual health challenges that present with anxiety. The LEAP involved in this project represents the opinions of a small group of individuals, who may not reflect those of a large proportion of the wider population, therefore impacting generalisability of the findings. Attempts to reduce this have been made by including people from diverse psycho‐social and cultural backgrounds.

## 5 Conclusion

The SAGE‐DM lived‐experience panels have established a global exemplar for participatory digital‐mental‐health research. They reframed risk, humanised data collection, and embedded inclusion as a methodological principle. As one participant summarised: “If this gives knowledge back to the people, that's fundamental. It's about empowerment, not extraction.” Their insights continue to shape the scientific and ethical architecture of the project, ensuring that SAGE‐DM is not just about detecting anxiety, it is about transforming how science listens.

## Author Contributions

**Sharon Lawn:** conceptualisation, investigation, methodology, writing – original draft, formal analysis, writing – review and editing, project administration, supervision, data curation. **Kerri M. Gillespie:** formal analysis, writing – review and editing, investigation, methodology. **Aakanksha Sahu:** investigation, methodology, writing – review and editing, formal analysis. **Stephanie J. Tobin:** methodology, investigation, writing – review and editing, formal analysis. **Vasundhara Shukla:** methodology, investigation, writing – review and editing, formal analysis. **Rachael Burns:** investigation, methodology, writing – review and editing, formal analysis. **Joanne Cockle:** investigation, methodology, formal analysis, writing – review and editing. **Amrita Dasvarma:** investigation, methodology, writing – review and editing, formal analysis. **Aislin Gleeson:** formal analysis, writing – review and editing, investigation, methodology. **John Milham:** investigation, methodology, writing – review and editing, formal analysis. **Robyn Priest:** investigation, methodology, writing – review and editing, formal analysis. **Puneet Sansanwal:** investigation, methodology, writing – review and editing, formal analysis. **Ashton Spradbury:** investigation, methodology, writing – review and editing, formal analysis. **Anna Zhang:** investigation, methodology, writing – review and editing, formal analysis. **Christine Kaine:** investigation, methodology, writing – review and editing, formal analysis, project administration. **Selena E. Bartlett:** conceptualisation, investigation, funding acquisition, writing – original draft, writing – review and editing, methodology, formal analysis, project administration.

## Ethics Statement

This study was approved by the Flinders University Human Research Ethics Committee (No.8983), with national mutual recognition by Queensland University of Technology (QUT) Research Governance and Integrity Team (No.10528). This included provision of a Participant Information Sheet to all LEAP members, with assurance that their participation was voluntary and that their specific contributions to LEAP discussions would be deidentified in any reporting. Given their core involvement across the project's research design, analyses and reporting stages, and with their permission, LEAP members were recognised as co‐authors.

## Consent

All LEAP co‐design workshop members provided their consent to be involved in the project.

## Conflicts of Interest

The authors declare no conflicts of interest.

## Supporting information

Supporting File

## Data Availability

The data that support the findings of this study are available on request from the corresponding author. The data are not publicly available due to privacy or ethical restrictions.
