# Supplementary material for: Co‐Designing a Social Media and Anxiety Survey: Reflections on the Importance of Centring Mental Health Lived Experience Expertise
Source: Health Expect. 2026 Apr 24;29(2):e70677. doi: 10.1111/hex.70677 (PMC13108405; doi:10.1111/hex.70677)
Supplement: Supplementary file 1 — Supporting File [file HEX-29-e70677-s001.docx]

**Supplementary File 1:**

**Lived Experience Co-Design Working Group**

**Guide Questions for Survey Development and Discussion**

**Please look through the draft survey and tell us what you think of:**

1. The draft survey questions:
   - Are the questionnaires too long?
   - Are the questions too confronting?
   - Do the questions make sense? Are there questions we have missed?
   - Should the order of the questions be changed?
   - Does the survey ask the most relevant questions from a lived experience perspective?
   - Other aspects of social media that make you feel anxious that we haven’t considered?
2. How would you feel about an app on your phone that would track your mood? (If you identify as a family supporter/carer, consider how the person you support might feel):
   - Do you think this would be beneficial?
   - In what situations would you be most likely to use this tool? (e.g. during stressful periods, daily check-ins, only when feeling anxious)?
   - What type of features would make you stop using this tool?
   - What do you think it should look like? (colours, format, content etc)
3. How would you feel about an app on your phone that would track your social media use?
   - Do you think this would be beneficial?
   - What do you think it should look like? (colours, format, content etc)
   - Would you want control over what data is collected and shared? What would this look like?
4. Our study may capture regular screenshots of your social media pages you are using. How would you feel about that?
5. What information would you want to have about an app that tracked your social media use (in order to use/trust it)?
6. Do you think an app like this would be effective in helping you change your use of social media / help with your anxiety?
7. Would you like the option to connect the app to support networks, e.g. sending a message to a trusted contact when distress is high?
8. Should the app include contact details for community or professional supports (what kind of supports?)
9. Should the app include other information? (what kind of information?)
10. What would make you feel safe, respected, and supported when using this tool?
11. Major concerns and practical issues relating to having your mood or social media tracked that you think are important from a lived experience perspective? (how do we address these?)
12. Do you have any suggestions for making the tool more helpful, trustworthy, and non-intrusive.
